# Genome-Wide Association Study in Rice Revealed a Novel Gene in Determining Plant Height and Stem Development, by Encoding a WRKY Transcription Factor

**DOI:** 10.3390/ijms22158192

**Published:** 2021-07-30

**Authors:** Xiaoshuang Wei, Hailian Zhou, Deying Xie, Jianguo Li, Mingchong Yang, Tianli Chang, Dongxin Wang, Lihua Hu, Guosheng Xie, Jihong Wang, Lingqiang Wang

**Affiliations:** 1 State Key Laboratory for Conservation & Utilization of Subtropical Agro-Bioresources, College of Agriculture, Guangxi University, Nanning 530004, China; 1917301040@st.gxu.edu.cn (X.W.); 2017401011@st.gxu.edu.cn (H.Z.); jianguo_li@webmail.hzau.edu.cn (J.L.); 1931200422@st.gxu.edu.cn (M.Y.); 2014401025@st.gxu.edu.cn (T.C.); 2 College of Plant Science & Technology, Huazhong Agricultural University, Wuhan 430070, China; xiedeying@126.com (D.X.); xiegsh@mail.hzau.edu.cn (G.X.); 3 College of Life Science & Technology, Guangxi University, Nanning 530004, China; biowangdongxin@163.com (D.W.); hulihua@gxu.edu.cn (L.H.); 4 Department of Life Science, Tangshan Normal University, Tangshan 063000, China; wjhongok@126.com

**Keywords:** OsWRKY21, rice, gibberellin, stem elongation, stress responses

## Abstract

Semi-dwarfism is a main agronomic trait in crop breeding. In this study, we performed genome-wide association study (GWAS) and identified a new quantitative trait nucleotide (QTN) for rice shoot length. The peak QTN (C/T) was located in the first coding region of a group III WRKY transcription factor *OsWRKY21* (LOC_Os01g60640). Interestingly, further haplotype analysis showed that C/T difference only existed in the *indica* group but not in the *japonica* group, resulting in significant differences in plant height among the different *indica* rice varieties. *OsWRKY21* was expressed in embryo, radicle, shoots, leaves, and stems. Most notably, overexpressing *OsWRKY21* resulted in the semi-dwarf phenotype, early heading date and short internodes compared to the wild type, while the knockout mutant plants by CRISPR/Cas9 technology yielded the opposite. The overexpressing lines exhibited the decreased length of the cells near sclerenchyma epidermis, accompanied with the lower levels of indole-3-acetic acid (IAA) and gibberellin 3 (GA_3_), but increased levels of the abscisic acid (ABA) and salicylic acid (SA) in the internodes at heading stage. Moreover, the semi-dwarf phenotype could be fully rescued by exogenous GA_3_ application at seedling stage. The RNA-seq and qRT-PCR analysis confirmed the differential expression levels of genes in development and the stress responses in rice, including GA metabolism (*GA20ox2*, *GA2ox6,* and *YABY1*) and cell wall biosynthesis (*CesA4*, *7,* and *9*) and regulation (*MYB103L*). These data suggest the essential role of *OsWRKY21* in regulation of internode elongation and plant height in rice.

## 1. Introduction

Rice (*Oryza sativa* L.) is a major staple crop worldwide that feeds more than half of the global population [1]. Semi-dwarfism is one of the most attractive traits in cereal crop breeding programs. Dwarf cultivars of many crop plants have been identified with enhanced lodging resistance, improved harvest index, and being responsive to fertilizer input [2]. The adoption of two well-known dwarf genes, semi-dwarf1 (*sd1*) in rice, and reduced height1 (*Rht1*) in wheat (*Triticum aestivum*), to create semi-dwarf varieties has significantly increased crop yields and initiated the “Green Revolution” [3]. At least 70 dwarf mutants have been discovered in rice, and some of them are involved in gibberellic acid (GA) biosynthesis or in GA-based signal regulatory pathways [4,5,6,7]. Since the plant height is typically quantitatively inherited, the genetic mapping and subsequent gene cloning by quantitative trait loci (QTLs) and/or genome-wide association study (GWAS) are ongoing to uncover more genes associated with the trait, despite many dwarf or semi-dwarf mutants and genes having been already reported.

The WRKYs is an important family of transcription factors that widely participate in plant development and stress responses. WRKY proteins contain the conserved WRKYGQK stretch in its N-terminus and a zinc-finger motif Cx_4–5_Cx_22–23_HxH or Cx_7_Cx_23_HxC in its C-terminus [8]. WRKYs can be classified into three groups, (I, II, and III), according to their structures. WRKY proteins regulate the transcription of the target genes by specific binding to W-box motifs ((T)TGAC(C/T)) in their promoter regions [9].

The past two decades have witnessed the extensive progresses in revealing the functions of plant WRKY transcription factors. The important roles of many *WRKY* genes in plant disease resistance or stress tolerance have been inferred from their overexpression- or knockdown- transgenic plants as well as their loss-of-function mutants [10,11,12,13]. *OsWRKY30* has a role in drought response in rice [11,14]. *WRKYs* were also reported to play diverse roles in plant growth and development [8,15]. For example, the *OsWRKY42* has a role in rice senescence [11,14]. In *Arabidopsis*, the disruption of *AtWRKY12* represses flowering, whereas loss of *AtWRKY13* promotes flowering [16]. Transparent Testa Glabra2 (TGG2, a WRKY transcription factor) and AtWRKY10 were reported to be involved in the regulation of seed grain size in *Arabidopsis* [17,18].

The rice genome has been predicted to contain 102 *WRKY* (*OsWRKY*) genes [19]. The WRKYs in rice are involved in regulating a range of biological processes involved in plant growth, development, and stress responses [20]. Several *WRKY* genes are expressed in response to the rice blast fungal elicitor1and the defense signal molecules salicylic acid (SA) and jasmonic acid (JA) [21]; among them the *OsWRKY13* and *OsWRKY45* are involved in SA-mediated defense signaling transduction in rice [22,23]. In addition, several studies have shown that WRKY proteins also participate in the regulation of plant growth and development in rice. OsWRKY71 can block response to gibberellin (GA) signal in aleurone cells by repressing *Amy32b* expression through specific binding to the W-box in the promoter region of *Amy32b* [24]. Knock-down of *OsWRKY78* results in a semi-dwarf phenotype and small seed grain size due to reduced cell length, whereas the accumulation of OsWRKY11 leads to semi-dwarf stature [25,26]. Recently, it was reported that OsWRKY36 inhibits GA signaling pathway thus represses both plant height and grain size [27]. Therefore, it can be concluded that the WRKY family members possess multiple functions in rice. In addition, the interrelations of GA biosynthesis, signaling with the WRKYs involved in regulating plant height and grain size remains to be elucidated.

In this study, to characterize the genetic basis of plant height, we firstly identified a consistent quantitative trait nucleotides (QTNs) for both shoot length and culm length with GWAS. The candidate gene encode a transcription factor belongs to group III WRKY subfamily in rice (OsWRKY21). Moreover, we performed a phylogenetic and structural analysis to predict its potential functions and explored its expression profile using multiple tissue samples throughout the development of rice. In addition, overexpressing *OsWRKY21* displayed a semi-dwarf and had a short cell near sclerenchyma epidermis in the internodes of stem. The OE lines showed decreased levels of endogenous IAA and GA_3_, and increased levels of ABA and JA in the stem internodes. These results, combined with the gene expression alteration in the transgenic plants via RNA-seq analysis, suggest that *OsWRKY21* is associated with the phytohormones metabolic pathway in the regulation of the plant development and the stress responses.

## 2. Results

### 2.1. Genome-Wide Association Study (GWAS) Identified the OsWRKY21 as a Candidate Gene for Plant Height in Rice

To identify the QTNs associated with the rice shoot length, we collected the phenotypic data from the seedling of 469 accessions (association panel) being cultivated under normal conditions in a growth chamber. The association panel exhibits extensive phenotypic variation in shoot length and the normal distribution of the trait, which are suitable for the GWAS analysis. Besides, QQ-plot indicated the accuracy of the GWAS result. The genotypes of the 469 accessions were downloaded from the website https://s3.amazonaws.com/3kricegenome/snpseek-dl/, accessed on 5 June 2021. Then, the original single nucleotide polymorphisms (SNPs)were filtered and finally 406, 858 high quality SNPs were obtained for genetic mapping. The average maker density was one SNP per 917 bp which is high enough for the accurate mapping of QTNs.

**Figure 1:**
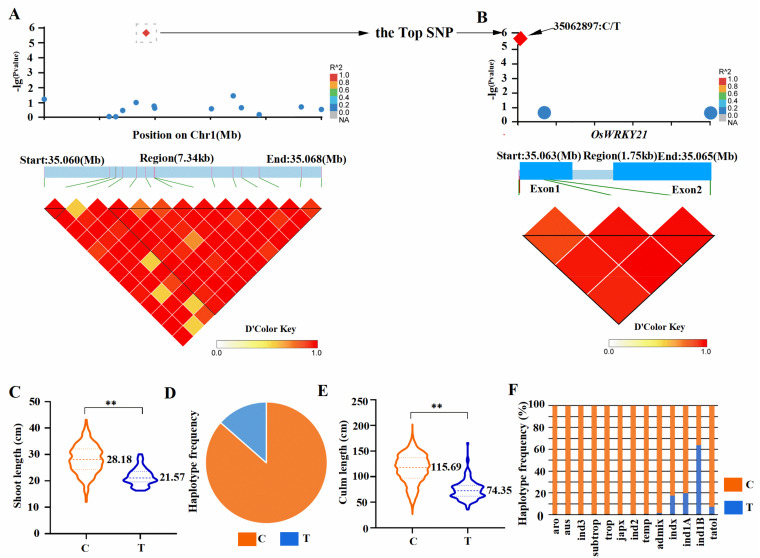
GWAS identification of candidate genes for shoot length at seedling stage (**A**–**D**) and culm length at heading stage (**E** and **F**). (**A**) SNPs at the region of 35060000-35368000 on Chromosome1. (**B**) GWAS identification of *OsWRKY21* as a candidate gene for shoot length at seedling with the group of Manhattan plots, candidate gene structure, and putative causal polymorphisms (C/T) (the position SNP C/T is indicated by an arrow). (**C**) Shoot length is significantly different between two different haplotypes C and T at *p* < 0.01 (** indict *p*-value < 0.01, *t*-test, *n* ≥ 20). (**D**) Haplotype frequency of C type and T type in the association panel. (**E**) Culm length is significantly different between two different haplotypes C and T at *p* < 0.01 (** indict *p*-value < 0.01, *t*-test, *n* ≥ 20). (**F**) Haplotype frequency of C type and T type in the different subpopulation of the association panel.

As a result, 511 SNPs were detected of shoot length on chromosomes 1, 2, 3, 4, 5, 6, 7, 8, 9, 10, and 12. Among them, one of the significant SNPs was found to be located at the region of 35060000-35368000 on Chromosome1 (Figure 1A). We further explored the region (Locus zoom in) and found that this top SNP was exactly located at the CDS coordinates (5′-3′): 35,062,734–35,064,940 on Chromosome 1, with the peak at chr1:35062897 (Figure 1B), which was the new QTN, never reported for plant height in any previous studies. This peak QTN located in the genic region of gene (LOC_Os01g60640), which encodes a protein annotated as the WRKY transcription factor (Figure 1B). The haplotype analysis revealed that there are two major haplotypes. The gene LOC_Os01g60640 (Os*WRKY21*) from the QTN in this region contains mainly two synonymous variants (T/C) at the locus chr1:35062897 located in the coding region (the first exon). Accessions with the variant allele cytosine (C) (haplotype C with mean value 28.18 cm) have higher shoot length than those with the allele thymine (T) (haplotype T with mean value 21.57 cm) (Figure 1C,D). When further investigated the haplotypes C and T in subpopulations ind1A and ind1B, we found that the average value of shoot length of the ind1A in haplotype C and haplotype T were 30.05 cm and 22.52 cm, respectively. While the average value of culm length of the ind1B in haplotype C and T were 23.28 cm and 21.12 cm, respectively.

Interestingly, when searching a QTN database at the website (https://snp-seek.irri.org/index.zul;jsessionid=56FCC6570012BB87A79B6FBC2 May 29, 20210CD995B, accessed on 29 May 2021), we found that a QTN for culm length at heading stage is in good agreement with our GWAS result in seedling stage (haplotype C with mean value 115.69 cm; haplotype T with mean value 74.35 cm) (Figure 1E,F). We further investigated the haplotypes C and T in subpopulations ind1A and ind1B and found that the average value of the culm length in haplotype C was 97.62 cm and that of the haplotype T was 65.12 cm in ind1A, while the average value of the culm length in haplotype C was 82.68 cm and that of the haplotype T was 73.11 cm in ind1B. Thus, this QTN within the *OsWRKY21* is expected to be associated with the plant height at both stages and worth being investigated further.

### 2.2. Bioinformatics and Expression Analysis of OsWRKY21 during the Development of Rice

**Figure 2:**
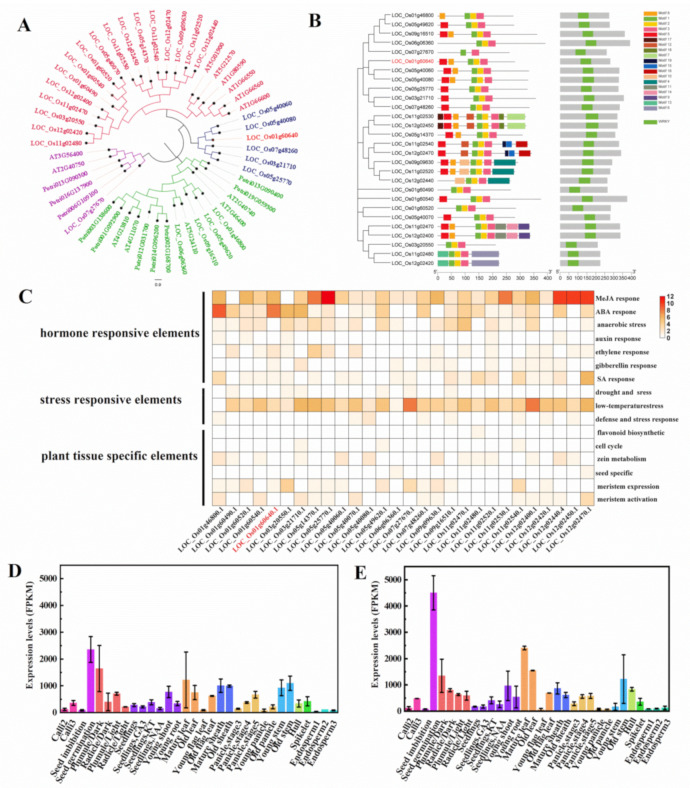
Structural analysis and expression pattern of the *WRKY* group III. (**A**) Phylogenetic analysis of WRKY group III among rice, *Arabidopsis* and poplar. GenBank accession number is preceded by a species identifier. Potri, *Populus trichocarpa*; AT, *Arabidopsis thaliana*; Os, *Oryza sativa*. (**B**) MEME motif analysis and conserved domain in OsWRKY group III. (**C**) Promoter analysis of OsWRKY group III. The horizontal axis shows the 28 genes of OsWRKY group III, and the vertical axis shows the corresponding cis- elements, the darker the color is, the more cis- elements represented. (**D**) and (**E**) The expression pattern of the *OsWRKY21* in the varieties Minghui63 and in Zhenshan97.

A phylogenetic analysis of WRKY families among rice, *Arabidopsis* and *Populus* showed most WRKY domains of the same type forming independent domains within their species, OsWRKY21 proteins was most closely related to the other five OsWRKYs and thus they were grouped in the same subclade (Figure 2A), suggesting a recent duplication within the clade occurred after the divergence of the monocotyledons from dicotyledons as reported in previous studies [28,29]. Based on amino acid sequence similarity, OsWRKY proteins were divided into three classes. Of which, class II WRKYs were divided into 10 subclasses (IIa-IIj), and class III WRKYs were divided into two subclasses (IIIa and IIIb). WRKY21 belongs to type IIIb, which carries a putative sumoylation site at the C-terminus and a potential coiled-coil domain at the N-terminus in addition to a conserved WRKY domain (WRKYGQ) and a zinc-finger-like motif (CX_6_CX_26_HX_1_C) (Supplementary Figure S1). A schematic representing the structure of OsWRKY group III proteins was constructed from the MEME motif analysis results (Figure 2B). Other than motifs 1 and 2 which are the WRKY domains widely distributed, OsWRKY members within the same clades were usually found to share a similar motif composition. Subcellular locations, molecular weight, isoelectric point, and transmembrane helices of OsWRKY group III proteins were seen in Supplementary Table S1. The promoter region cis-elements determine the temporal and spatial expression of the genes [30]. To understand the transcriptional regulation of the *OsWRKY* group III genes, the cis-elements in the promoter regions of those genes were identified using the PlantCARE database. These elements could be categorized into three classes which are hormone responsive, stress responsive, and tissue specific (Figure 2C; Supplementary Table S2). Among them, the group of hormone-responsive elements is the largest one and the group of stress-responsive elements the second. These results suggested that *OsWRKY* group III genes could be regulated by both the environmental and developmental changes, implying their roles in physiological processes and developmental events.

To observe the expression pattern of the *OsWRKY21*, microarray datasets from 33 tissues across the whole life cycle of rice were downloaded from CREP database (Collections of Rice Expression Profiling, http://crep.ncpgr.cn, accessed on 18 November 2020) [31]. Overall, *OsWRKY21* was expressed widely in many different tissues examined during plant growth and development, although the expression levels varied greatly (Figure 2D,E; Supplementary Table S3). In detail, the *OsWRKY21* was mainly expressed in the embryo and radicle after germination, the plumules and radicles, the shoots at the seedling stage, as well as the leaves and stems at the heading stage, with weak expression observed in calli and younger endosperms (Figure 2D,E). Notably, the expression of the *OsWRKY21* was dramatically enhanced in the embryo and radicle after germination compared to the seeds 72 h after imbibition (before germination). Secondly, the *OsWRKY21* is likely to be inhibited by light as its expression was much lower in both plumules and radicles exposed to light than those in dark. Thirdly, the expression of *OsWRKY21* was much stronger in both leaves and stems at heading stage than those before the heading stage, indicating its higher and preferred expression in the tissues undergoing secondary growth (elongation of stems).

### 2.3. OsWRKY21 Overexpression Resulted in Semi-Dwarf Phenotype While the Knockout Mutant Plants by CRISPR/Cas9 Technology Yielded the Opposite, Which Confirms the GWAS Results

**Figure 3:**
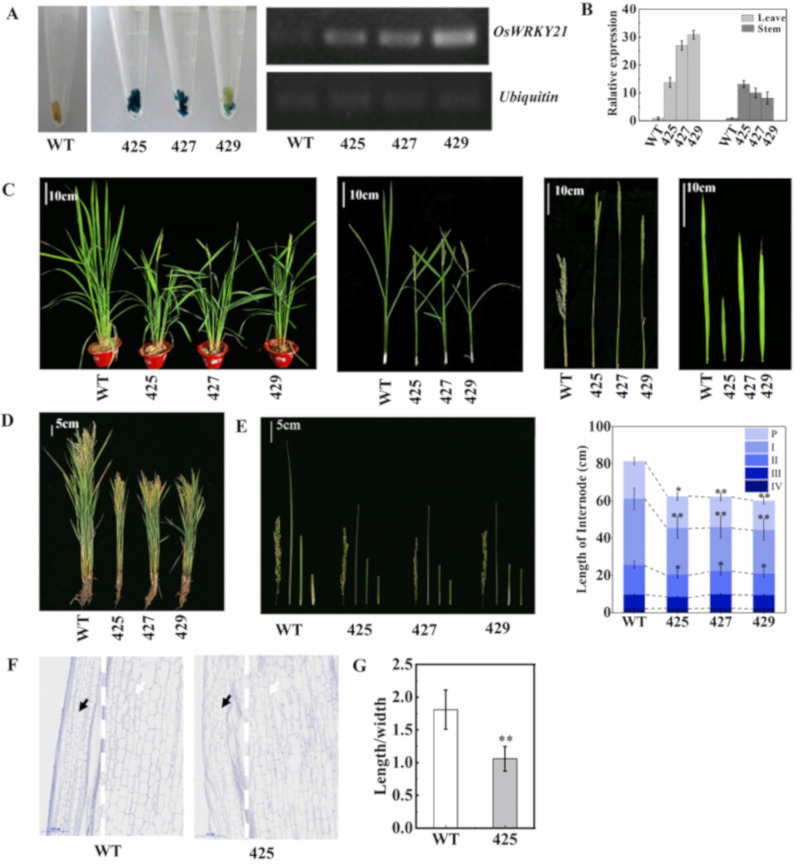
Generating and Characterization of *OsWRKY21* transgenic plants**.** (**A**) GUS staining and semi-quantitative RT-PCR to select positive transgenic lines (425, 427 and 429). The positive *OsWRKY21* OE lines were in blue with the GUS staining. Gene Ubiqutin was used as the internal control in RT-PCR analysis. (**B**) Relative expression of *OsWRKY21* in the wild-type ZH11 and the OE plants using qRT-PCR. (**C**) Phenotypes of the wild-type ZH11 and three OE lines (425, 427 and 429) at heading stage, scale bar = 10 cm. (**D**) and (**E**) Lengths of the panicles and internodes of wild-type ZH11 and OE plants at maturity stage. P, panicle; I, the uppermost internode; II, III, IV, the second, third, and fourth internodes counted from the up to bottom, respectively. (* and ** indict *p*-value < 0.05 and 0.01, respectively, *t*-test, *n* = 15). (**F**) and (**G**) Comparison of the longitudinal sections of the second uppermost internodes of WT and line 425, scale bar = 200 μm. Values of the ratios of cell length to width of WT and 425 line, values are means ± SD of 20 cells (* *p* < 0.05, ** *p* < 0.01, *t*-test, *n* = 20).

As clearly showed by the GWAS result and the expression analysis, the OsWRKY21 was expected to be involved in rice plant growth. To validate this expectation and to further study the function of *OsWRKY21*, the CDS of the gene was cloned from the cDNA of the japonica rice cultivar Zhonghua 11 (ZH11) and inserted into the destination binary vector pTCK303. The construct carrying the CDS of *OsWRKY21* (OE-pTCK303-WRKY21) was generated and was introduced into the ZH11 via *Agrobacterium*-mediated transformation. The positive transgenic lines were selected based on both RT-PCR and GUS staining (Figure 3A), and further confirmed by sequencing analysis (data not shown). After three generations, three independent homozygous rice transgenic lines (T_3_ plants named 425, 427, and 429, respectively) were selected. The qRT-PCR results clearly indicated that the expression levels of *WRKY21* were increased significantly at level of 0.01 in both culms and leaves in all three OE lines compared with wild-type ZH11 plants (Figure 3B). Thus, these independently transgenic plants overexpressing *OsWRKY21* were used for further investigation.

When grown in the field, all plants of OE lines showed a semi-dwarf phenotype, with plant height about three-quarters of that of the wild-type ZH11 (Figure 3C). Compared to the wild-type, the lengths of the internodes of the OE plants were significantly reduced (Figure 3D, E). To investigate whether the reduced length of panicles and internodes in the OE lines is due to the defects in cell elongation or cell proliferation, we compared the longitudinal sections of the elongated zone of the uppermost internodes from OE and wild-type plants. Microscopic observation showed that the length of the cells near sclerenchyma epidermis in the longitudinal direction was shorter in OE line 425 than that in wild-type ZH11 (Figure 3F,G), indicating that overexpression of *OsWRKY21* mainly impaired the elongation of the internode. That is the same in OE line 427 and 429 (data not shown). It is worth mentioned that the length of the leaves also decreased, whereas the width of leaves remains unaffected, again illustrating that the gene mainly affect the elongation of cells.

### 2.4. OsWRKY21 Suppressed IAA and GA Levels and the Dwarf Phenotype Could Be Rescued by Exogenous GA Treatment

**Figure 4:**
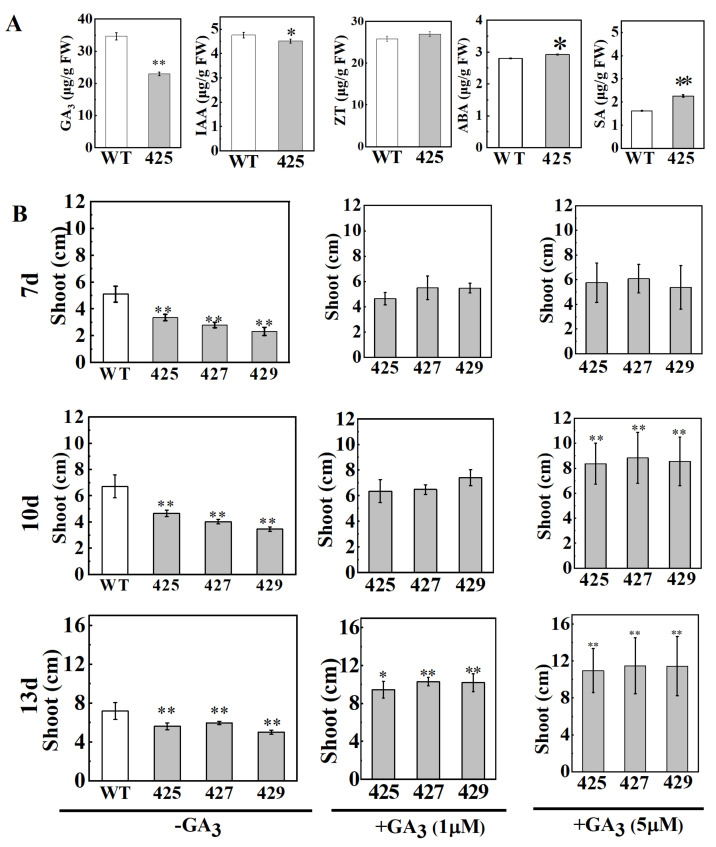
The hormones changes in OE plants and semi-dwarf phenotype could be Rescued by Exogenous GA_3_ Treatment. (**A**) Contents of endogenous hormones GA_3,_ IAA, ZT, ABA, and SA in the stems from the wild-type ZH11 and OE line plants (* *p* < 0.05, ** *p* < 0.01, *t*-test, *n* = 15). (**B**) wild-type ZH11 and three OE lines (425, 427, and 429) treated without or with 1 μmol/L GA_3_ and 5 μmol/L at seedling stage (Values are means ± SD, * *p* < 0.05, ** *p* < 0.01, *t*-test, *n* = 15).

The growth-promoting hormones such as IAA and GA regulate diverse developmental processes throughout the life cycle of the plants. To our knowledge, low levels of the hormones or the disruption of the hormone signaling pathways can cause the dwarf of the plant. Thus, we investigated the levels of the two kinds of phytohormones in the OE line 425 and found that the levels of endogenous GA_3_ and IAA were significantly decreased in the OE plants compared to that of the wild-type ZH11, respectively (Figure 4A). Interestingly, we also found that the levels of stress-related hormones abscisic acid (ABA) and salicylic acid (SA) were increased in the stem internodes.

To confirm whether the semi-dwarf phenotype of *OsWRKY21*-OE plants is caused by GA deficiency, we investigated the response of OE plants to exogenous GA at seedling stage. We found that without the GA_3_ treatment, all plants of the three OE lines exhibit obviously shorter shoots than the wild type ZH11 at early seedling stage (Figure 4B; Supplementary Figure S2). While continually treated with GA_3_ from the germination stage, all OE plants showed normal elongation but slightly slender (Figure 4B; Supplementary Figure S2). Exogenous application of GA_3_ had obvious effects on promoting the growth of all OE lines seedlings but had no obvious impact on the growth of the wild-type ZH11. Thus, it was evident that the semi-dwarf phenotype of *OsWRKY21* OE lines is associated with the decreased endogenous GA_3_ level, which could be fully rescued by exogenous GA_3_ application.

### 2.5. Overexpression of OsWRKY21 Altered the Expression Levels of the Genes for Phytohormones Metabolic Pathway and Secondary Cell Wall Biosynthesis

**Figure 5:**
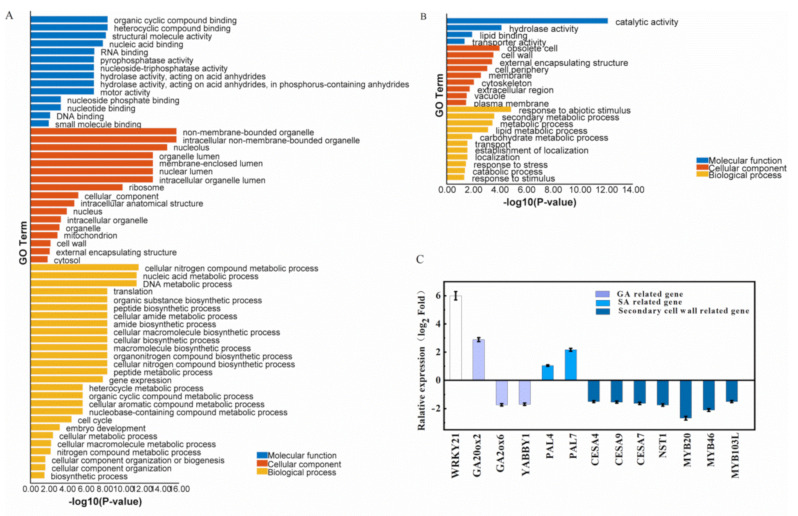
GO analysis of differentially expressed genes upregulated (**A**) or downregulated (**B**) in *OsWRKY21* OE plants compared to wild–type ZH11; (**C**) the expression of the GA, SA metabolic genes and the typical secondary cell wall biosynthesis and regulatory genes.

To confirm the involvement of *WRKY21* as a negative regulator in the level of GA and stem elongation in secondary tissue growth stages, we examined the global gene expression in the second internode of the OE line 425 and of the wild-type ZH11 via RNA-seq analysis. The genes with two-folds changes in expression and with the significant *p*-value were subjected to GO enrichment analysis (Figure 5A,B). The expression of the gene involving in the cellular biosynthetic and cell cycle were up-regulated, while the gene of secondary metabolism and response to stress were down-regulated. As expected, the expression of the OsWRKY21 was dramatically increased in the OE plants, six-fold more than that of the wild-type ZH11, consisting with the qRT-PCR result (Figure 2B and Figure 5C). Altered expression of the genes involved in phytohormones metabolic pathways such as those for the biosynthesis of GA and SA was observed. The genes for gibberellin 2-beta-dioxygenase 7 (LOC_Os04g44150) and *YABBY1* (LOC_Os07g06620) were decreased, whereas the genes for gibberellin 20 oxidase 2 (LOC_Os05g34854) were increased (Figure 5C). It was reported that either overexpression or co-suppression of *OsYABBY1* impaired GA-mediated repression of *GA3ox2* [32]. The overexpression of the rice *YABBY4* gene (*OsYABBY4*) leads to a semi-dwarf phenotype, abnormal development in the uppermost internode [33]. In addition, the expression levels of many genes for development were decreased (Figure 5B,C). Notably, the expression of the genes that encode three cellulose synthases *OsCESA4, -7* and *-9* (LOC_Os01g54620, LOC_Os10g32980 and LOC_Os09g25490), all of which being organized as rosette complex to biosynthesize the cellulose during the secondary cell wall formation were jointly decreased [34]. Interestingly, we also found that the expression of several transcriptional factors involved in the cell wall regulatory network, such as *MYB103L* (LOC_Os08g05520) were also decreased. Therefore, it was concluded that the overexpression of the *WRKY21* disturbed many aspects of plant growth and development.

## 3. Discussion

Phenotype–genotype association analysis has become a critical tool for identifying alleles and loci responsible for the agronomically important traits [35]. In the current study, the selection of rice accessions from diverse origins, with sufficient genetic variation and favorable population structure, is advantageous for GWAS implementation [36,37]. We identified a region harboring a gene which was never reported for plant height previously. Although the QTN effect of the *QsWRKY21* is not large, it can be consistently detected as significant at two different stages (seedling and heading). Thus, it strengthened our expectation that the *OsWRKY21* is the candidate gene for plant height. Firstly, we overexpressed the *OsWRKY21* in same genetic background Zhonghua 11 and the results clearly showed the semi-dwarf phenotype of the transgenic plants. However, considering that the early flowering and dwarf might be caused by the side effects of possible over-expression of transgene, we further created the knockout mutant plants by CRISPR/Cas9 editing which yielded the significantly increased plant height. Using CRISPR/Cas9 editing technology, this study selected two specific regions in exon 1 of the *OsWRKY21*, with the 20-bp target site for the design of a sgRNA using CRISPR-P program (Supplementary Figure S3A). The binary constructs carrying the sgRNA within target regions with Cas9p driven by UBIp were generated (UBIp: Cas9-Os*WRKY21*) and transformed into the rice Zhonghua 11 via agrobacterium-mediated transformation and two independent lines were further sequenced to verify gene editing occurrence in this study (Supplementary Figure S3B). Interestingly, compared to the wild-type, the lengths of the internodes of the editing plants were significantly increased and obviously in the uppermost internodes (Supplementary Figure S3C). Thus, the overexpression of the *OsWRKY21* resulted in semi-dwarf phenotype while the knockout mutant plants by CRISPR/Cas9 editing yielded the opposite. The results of transgenic experiments strongly confirm the GWAS results and the roles *OsWRKY21* in plant growth. Our results indicated that the GWAS based on SNP marker density for agronomic traits in 469 rice lines was very efficient for the identification of new genes in plant height in rice.

### 3.1. OsWRKY21 Shows Multiple Functions in Growth/Development and Stress Responses in Rice

This study focused on the stem phenotype; however, the seed-setting rate of the OE lines were also found to be affected (data not shown). The changes in grain development and in seed germination warrant further investigations. *OsWRKY21* could have effects as early as the germination stage as the dwarf phenotype could be observed from the germination to the seedling stage. Since the GA is a kind of major hormone at germination stage, the dynamic changes in hormone contents and the gene expression of the Os*WRKY21* OE plants during the germination should be further investigated to elucidate the regulatory pathway of the Os*WRKY21*. In addition, we observed that large amounts of the genes involved stress response were up-regulated in Os*WRKY21* OE plants and found that the elements relevant to stress response were enriched in the promoter region of the *OsWRKY21* (Figure 2C and Figure 5A). Furthermore, we found that the stress-related hormones ABA and SA in the stem internodes were increased in Os*WRKY21* OE plants. The RNA-seq analysis support that the *OsWRKY21* functions in the regulation of the metabolic pathways of these phytohormones for their homeostasis. More recently, it was reported that *OsWRKY21* and *OsWRKY108* function redundantly to promote phosphate accumulation through maintaining the constitutive expression of OsPHT1 [38,39,40,41,42]. Collectively, these results suggest that the *OsWRKY21* probably is a master regulator of physiological process in development and stress responses. Therefore, the elaborate illustration of OsWRKY21 will be expected to regulate growth/development and environmental stresses in the future.

### 3.2. Semi-Dwarf Phenotype of OsWRKY21 Overexpression Is Attributed to the Integral Effects of Phytohormones in Rice

The WRKY transcription factor family genes were investigated to function in developmental processes and stress responses in plants; however, the function of the *WRKY21* is not well understood in monocotyledon. In this study, we showed that the *OsWRKY21* is a transcriptional repressor of plant stem development. *OsWRKY21* is expressed in almost all tissues of rice plants, and highly expressed in shoots and young stems. Overexpression of *OsWRKY21* resulted in the semi-dwarf phenotype, accompanied by the decrease of the phytohormones IAA and GA, which can be rescued by exogenous GA_3_ application. The subsequent RNA-seq analysis indicated the role of *OsWRKY21* in phytohormones metabolism including the negative regulation of the GA biosynthesis. However, further studies will clarify whether the Os*WRKY21* has the negative regulation effect on the endogenous hormones. Interestingly, the expression levels of those genes involved in secondary cell wall cellulose biosynthesis and regulation were significantly decreased in *WRKY21* OE lines. Interestingly, previous researches showed that the disruptions of these genes caused the semi-dwarf phenotype in plants [38,39,40,41]. Taken together, the semi-dwarf phenotype of the overexpression of *OsWRKY21* may be attribute to the integrated effects of the phytohormones.

## 4. Materials and Methods

### 4.1. Population Structure and GWAS

Genetic variation (single nucleotide polymorphism (SNP)) data for the 469 accessions were downloaded from (https://s3.amazonaws.com/3kricegenome/snpseek-dl/, accessed on 5 June 2021) [43]. We identified 406,858 SNPs after removing SNPs with minor allele frequencies 0.05, missingness per marker 0.02, and missingness per individual 0.01 by Plink [44]. The general linear model (GLM), mixed linear model (MLM), and compressed MLM model (CMLM) were used to analyze the genotyping data by rMVP and GAPIT [45,46].

### 4.2. Bioinformatics Analysis

The full-length protein sequences of WRKY group III in *Oryza sativa, Arabidopsis thaliana*, and *Populus L* were aligned by ClustalW. The unrooted phylogenetic trees were constructed using the Neighbor Joining (NJ) method with the following parameters: p-distance model, pairwise deletion and 1000 bootstrap replicates by MEGA7 software (http://www.megasoftware.net/, accessed on 10 November 2020) with 1000 bootstrap replicates [47]. The conserved motifs in the OSWRKY group III protein sequences were found using Multiple Expectation Maximization for Motif Elicitation (MEME) program version 4.0 (http://meme-suite.org/tools/meme, accessed on 20 November 2020) with the following parameters: number of repetitions, any; maximum number of motifs, 25; optimum motif width set to >6 and <200 [48]. Then conserved domains were analyzed by using Conserved Domains Database (CDD) (http://www.ncbi.nlm.nih.gov/cdd/, accessed on 20 November 2020). The protein of OSWRKY group III transmembrane helices were predicted using TMHMM2.0 (http://www.cbs.dtu.dk/services/TMHMM-2.0/, accessed on 20 November 2020) [49]. The subcellular locations were analyzed using WoLF PSORT (http://psort.nibb.ac.jp/, accessed on 5 November 2020) [50]. Prediction of molecular weight, isoelectric point was conducted using (http://cn.expasy.org/tools/pi_tool.html, accessed on 15 November 2020). The Plant CARE online software (http://bioinformatics.psb.ugent.be/webtools/plantcare/html/, accessed on 16 November 2020) was used to predict the promoter element and function of the OSWRKY group III gene [51]. Three-dimension structure of OsWRKY21 was performed using SWISS-MODEL online prediction software (http://swissmodel.expasy.org/, accessed on 16 November 2020).

### 4.3. Expression Analysis of OsWRKY21 in Rice

The expression profile data of *OsWRKY21* in 33 tissue examples (Supplementary Table S3) of Zhenshan 97 (ZS97) and Minghui 63 (MH63) were obtained from the CREP database (http://crep.ncpgr.cn, accessed on 18 November 2020) generated by a rice transcriptome project using the Affymetrix Rice GeneChip microarray [31].

### 4.4. Plasmid Vector Construction and Rice Transformation

The entire coding sequence (CDS) region (834 bp) of OsWRKY21 was amplified use the cDNA extracted from the Zhonghua 11 (ZH11) as the template. The forward primer is CGGGATCCTCCCAAGCTGAGAGTTGTCG (with *BamH* I site) and the reversed primer is GACTAGTCGTGCGATTATCT GACGAACT (with *Spe* I site). The fragment was first cloned into TA clones and sequenced to confirm its right sequence of the CDS. Then the CDS of the *OsWRKY21* was cloned into the destination vector pTCK303 to generate the overexpress construct OX-pTCK303-*WRKY21*. The construct carrying the CDS of the *OsWRKY21* was introduced into Agrobacterium tumefaciens strain EHA105 and transformed into the wild-type Zhonghua 11 as described previously [52]. The positive transgenic lines were selected based on PCR, GUS staining, and sequencing analysis.

The CRISPR plasmid vector pRGEB32 was used to transiently express U3p:sgRNA along with UBIp:Cas9 in rice. The construct carrying the U3p:sgRNA and UBIp:Cas9 was transformed into the Agrobacterium strain (EHA105) as described by Xie et al. [53]. The positive transgenic lines were selected based on PCR and sequencing analysis. The sequences of sgRNA (*OsWRKY21*) and the primers used in this study were listed in Table S4.

### 4.5. Plant Materials, Growth Conditions, and GA_3_ Treatment

Rice (*Oryza sativa* L.) Zhonghua 11 (*japonica* cv. ZH11) plants were grown in an experimental field at Huazhong Agricultural University (Wuhan, China) during the rice growing summer season Year 2020. All harvested seeds were dried at 40 °C for three days to break possible dormancy first. Then 15 seeds from each type of OE, and ZH11 plant were placed into beakers and soaked with tap water at room temperature at 25 °C for three days to ensure the complete absorption of the water, then subjected for GA_3_ treatment at levels of 0 μM and 1 μM, respectively throughout the stages of germination and seedling. After germination, seeds were placed on double sheets of filter paper in a 9 cm diameter Petri dish, moistened with water, and maintained at 30 °C and 60% relative humidity for 3 days seedlings were grown in the same conditions for another 7 d, and pictures of the representative plants were taken before and after the treatment using a Nikon D7000 camera. The lengths of seedlings were measured both before and after GA treatment. The experiments were carried out in three biological repeats.

### 4.6. RNA Extraction and qRT-PCR

Total RNAs were isolated from different tissues as indicated using a Hipure plant RNA Mini Kit, and the first-strand cDNA was synthesized from 2 μg of total RNAs using a PrimeScript™ RT reagent Kit from TaKaRa (code: RR047A, Beijing, China). The experiments were performed following the manufacturer’s instructions. SYBR-based qPCR (GoTaq^®^ qPCR Master Mix Kit, Promega code: A6002, California, United States) was set up in a reaction volume of 10 µL, and run with three replicates on a LightCycler 480 system (Roche, Basel, Switzerland) using the following reaction conditions: 95 °C for 1 min followed by 50 cycles of 95 °C for 10 s and 60 °C for 30 s. The rice ubiquitin gene was used as the internal control, and relative expression levels of genes were calculated using the 2^–ΔΔCt^ method [54]. Primers used in quantitative real-time PCR (qRT-PCR) are listed in Supplementary Table S4. Three biological replications were performed.

### 4.7. Measurement of Endogenous Phytohormones IAA and GA_3_

At the plant heading stage, the 2nd internodes from the plants of OE lines and wild type ZH11 were chosen for measurement of plant hormones IAA and GA_3_. A sample amount of 0.2 g was ground in liquid nitrogen with a mortar and pestle, then added with 1 mL of precooling 70–80% methanol solution (pH = 3.5), kept at 4 °C for overnight, then was centrifuged at 4 °C 12,000× *g* for 10 min and the supernatants were collected. The remaining residue soaked in 0.5 mL 70–80% methanol solution for 2 h at 4 °C then centrifugated as before and the supernatants were collected. The supernatants were combined and dried under vacuum at 40 °C to remove the organic solvents (the volume is 1/3 of the original). The equal volume petroleum ether was added and mixing, the samples were stood for a while for phase separation. The separation process of extracting and decoloring was repeated 2–5 times. Then, the triethylamine was added and the pH value was adjusted to 8.0, followed by the addition of PVPP (polyvinylpyrrolidone), and shaken while incubated for 20 min. The samples were centrifuged and the supernatants collected, then the hydrochloric acid was added to adjust the pH to 3.0. Extracting was performed by a separation process with ethyl acetate 3 times. The organic portions in the ester phase were combined and dried under vacuum at 40 °C, then were dissolved by adding the mobile phase solution and vortex blending and were filtered through a needle filter prior to chromatographic analysis.

HPLC analysis was performed in a LC-20AT (Shimadzu, Japan) equipped with an ultraviolet detector SPD-20A and a column temperature chamber CTO-20AC. The operating conditions were as follows: a C18 reversed-phase column (150 mm × 4.6 mm, 5 μm), mobile phase A: 100% methanol; B: Aqueous 0.1% acetic acid solution, A: B = 55:45; the injection volume 20 μL; the flow rate 0.8 mL/min; the column temperature 30 °C; detection was performed at 254 nm.

### 4.8. Histological Analysis

For microscopic observation, sections of the 2nd internode of plants at the heading stage were fixed in formaldehyde:glacial acetic acid:70% ethanol (1:1:19 *v/v/v*), softened by 15% hydrofluoric acid for 2 weeks, and then dehydrated in a graded ethanol series. The samples were embedded in paraffin wax (melting point: 56–58 °C). Microtome sections of 15 µm thicknesses were stained with toluidine blue and paraffin was removed using xylene before observation. Then imaged using a light microscope (BX-61, Olympus). The lengths and widths of cells (n ≥ 50) were measured using the ImageJ 1.32j software (https://imagej.nih.gov/ij/, accessed on 30 November 2020)

### 4.9. Analysis of the Sequencing Data

The raw sequencing data in the format of FastQ were quality-controlled using the FastQC (version 0.11.5, http://www.bioinformatics.babraham.ac.uk/projects/fastqc/, accessed on 1 November 2020) by removing the low quality reads and adaptor reads. Then, the remaining clean reads were mapped to the MSU7 version of the rice reference genome (http://rice.plantbiology.msu.edu, accessed on 5 November 2020) using hisat2 (version 2.1.0) [55]. Gene expression levels were quantified by FeatureCounts [56]. The DESeq (http://bioconductor.org/packages/release/bioc/html/DESeq.html, accessed on 7 November 2020) was used to identify the DEGs by pairwise comparison [57]. DEGs were identified using the Negative binomial distribution test with the criteria of *p* value < 0.05 and log2(Fold Change, FC) ≥ 1. Up-and down-regulated DEGs were identified as log_2_FC > 1 and log2FC < −1, respectively. The DEGs were further annotated with GO functional and KEGG pathway analyses using the software TBtools [58]. 

## Data Availability

Not applicable.

## Supplementary Materials

The following are available online at https://www.mdpi.com/article/10.3390/ijms22158192/s1.
