# Supplementary material for: Genome-Wide Association Study in Rice Revealed a Novel Gene in Determining Plant Height and Stem Development, by Encoding a WRKY Transcription Factor
Source: Int J Mol Sci. 2021 Jul 30;22(15):8192. doi: 10.3390/ijms22158192 (PMC8347446; doi:10.3390/ijms22158192)
Supplement: Supplementary file 1 [file ijms-22-08192-s001.zip › Supplementary figure.pdf]

Supplementary Figure S1

A

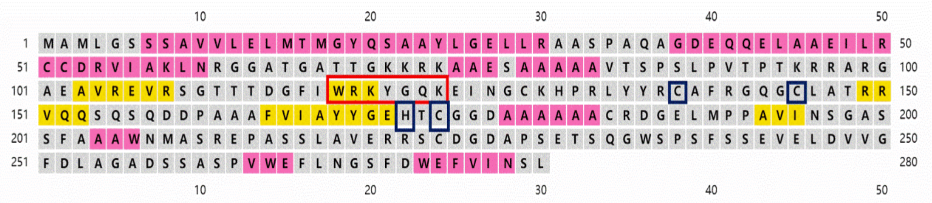

B

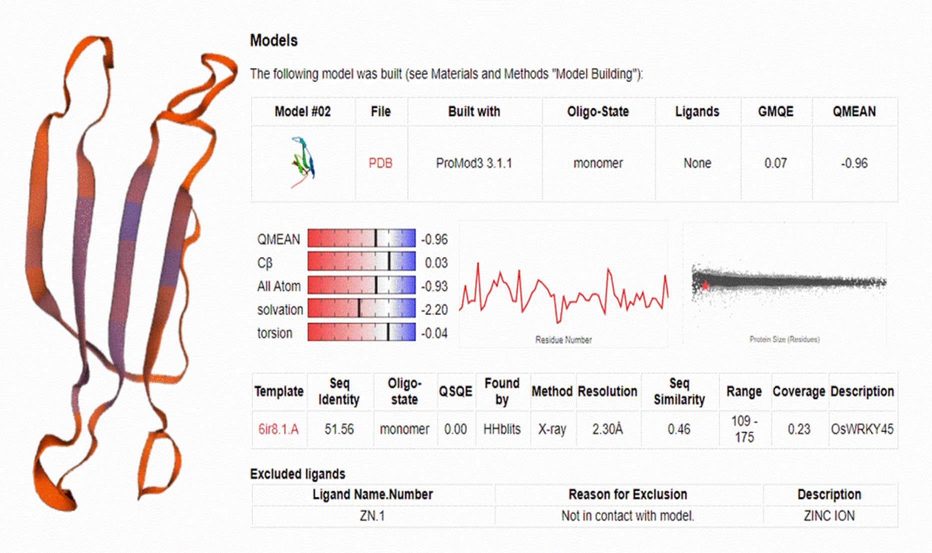

Supplementary Figure S1: Secondary and tertiary structures of OsWRKY21 protein

Predicted secondary structure of OsWTRKY21 protein (1A). Rectangles with different colors represents protein secondary structure, pink： helix; yellow: strand; grey: coil. The sequences of the OsWRKY21 motif (WRKYGQK) and the C2HC domain (CX6CX26HX1C) are highlighted by the red and blue rectangles, respectively (1A). Predicted tertiary structure of OsWRKY21 contained a conserved zinc-finger structure widely distributed WRKY family (1B).

Supplementary Figure S2

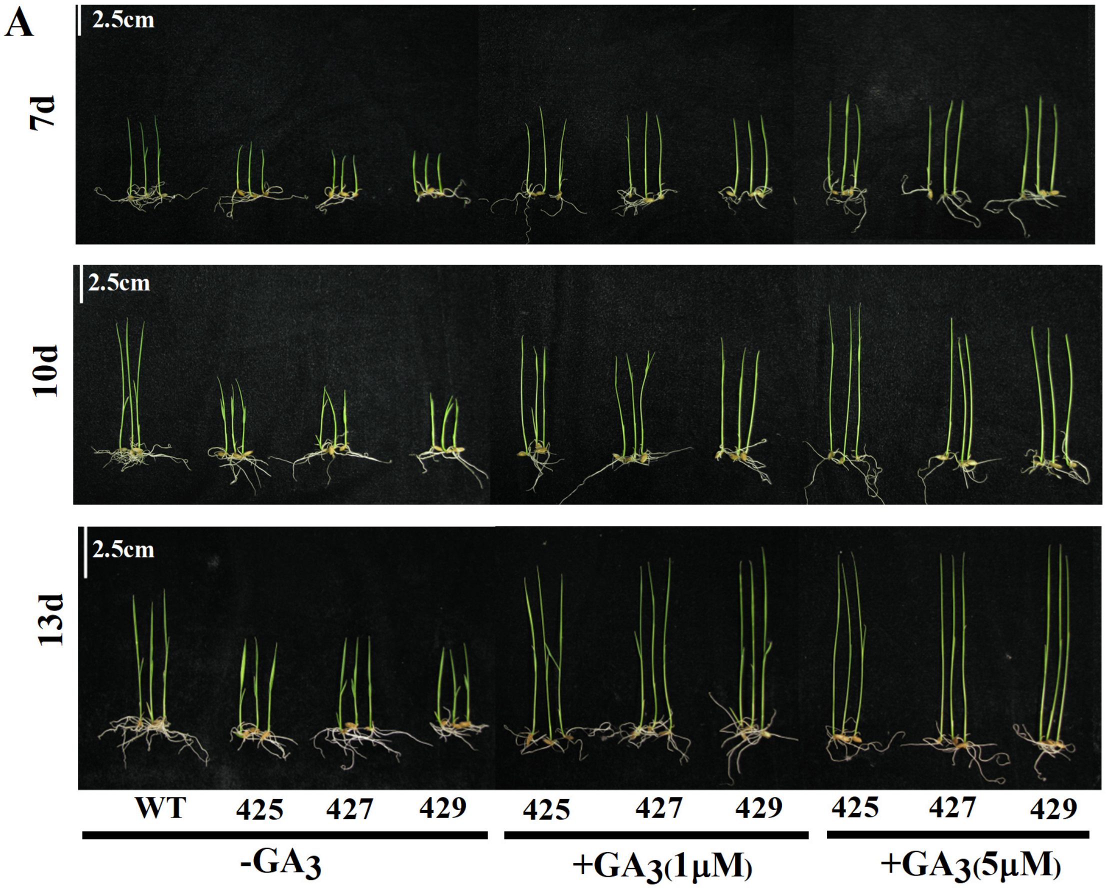

Supplementary Figure S2: Phenotype of wild-type ZH11 and three OE lines treated without or with GA_3 at seedling stage

Phenotype of wild-type ZH11 and three OE lines (425, 427 and 429) treated without or with 1 μmol/L GA_3 and 5 μmol/L at seedling stage

Supplementary Figure S3

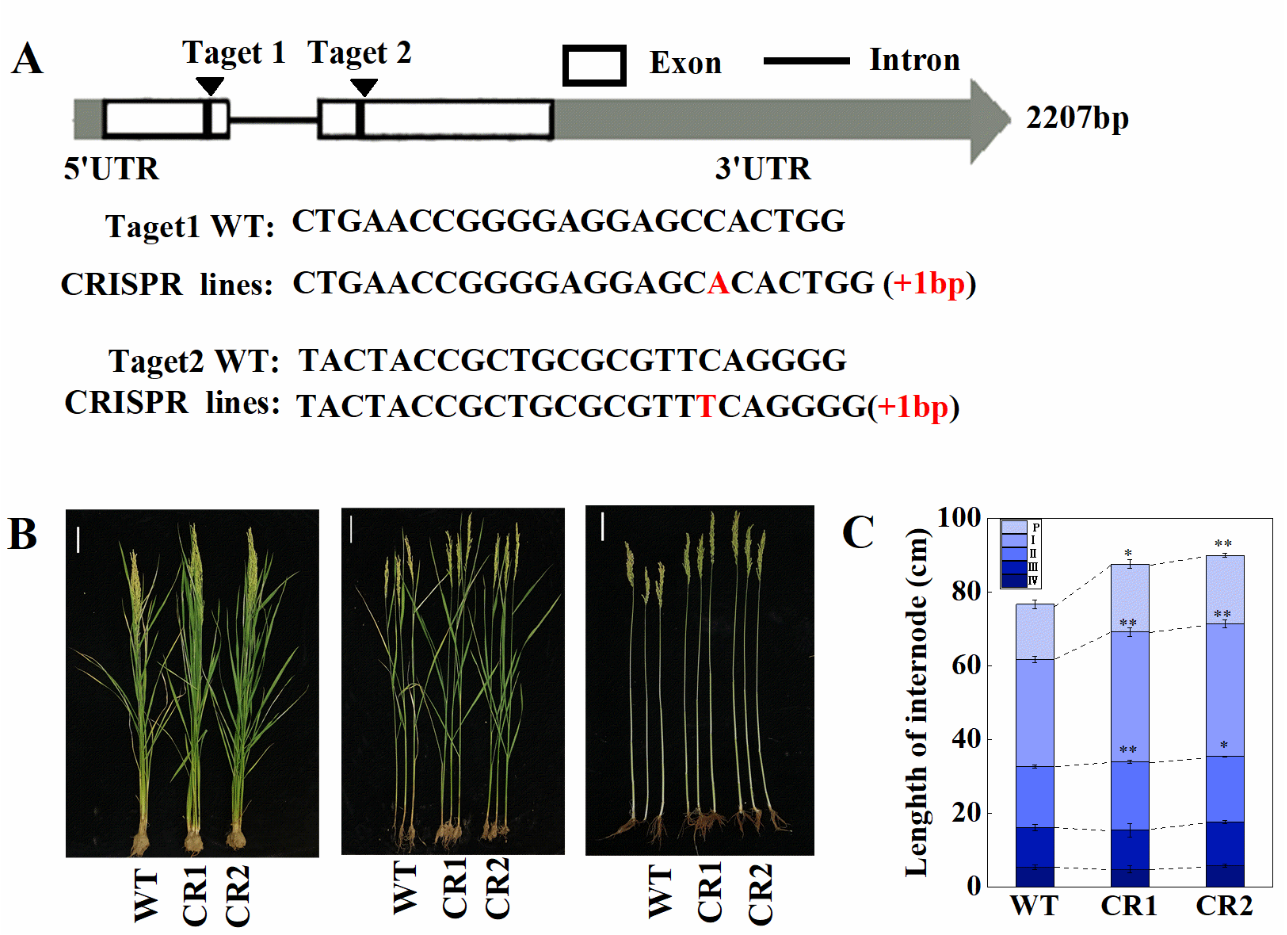

Supplementary Figure S3: *OsWRKY21* gene editing site information and phenotype

Construct for editing of target genomic sites in rice and PCR detection of target sequences in two independent lines. The *OsWRKY21* gene model includes one exons separated by two introns (represented by the solid line) and Red mark as mutation site. (3A); Phenotype of wild-type ZH11 and two CRISPR lines (CR1 and CR2), bar-= 25cm (3B) ;Lengths of the panicles and internodes of wild-type ZH11 and knockout mutant plants at heading stage. P, panicle; I, the uppermost internode; II, III, IV, the second, third, and fourth internodes counted from the up to bottom, respectively. (* and ** indict p-value < 0.05 and 0.01, respectively, t-test, n = 15)(3C).
